# A Review of Polymer-Stabilized Ferroelectric Liquid Crystals

**DOI:** 10.3390/ma7053568

**Published:** 2014-05-06

**Authors:** Ingo Dierking

**Affiliations:** School of Physics and Astronomy, University of Manchester, Schuster Building, Oxford Road, Manchester M13 9PL, UK; E-Mail: ingo.dierking@manchester.ac.uk; Tel.: +44-161-275-5067

**Keywords:** liquid crystal, polymer network, ferroelectric, antiferroelectric, photopolymerization, material properties

## Abstract

The polymer stabilized state of ferroelectric liquid crystals (FLC) is reviewed; and the effect of a dispersed polymer network in an FLC outlined and discussed. All fundamental material aspects are demonstrated; such as director tilt angle; spontaneous polarization; response time and viscosity; as well as the dielectric modes. It was found that the data can largely be explained by assuming an elastic interaction between the polymer network strands and the liquid crystal molecules. The elastic interaction parameter was determined; and increases linearly with increasing polymer concentration.

## 1. Introduction

### 1.1. Liquid Crystals [1–3]

Liquid crystals are anisotropic fluids, which are thermodynamically located between the isotropic liquid and the three-dimensional crystal. Two broad classes of liquid crystals are distinguished, lyotropic phases [4,5], which are formed by variation of the concentration of amphiphilic molecules in a suitable solvent, and thermotropic phases, which are observed by temperature variation. The former class of liquid crystals will be disregarded in this review, while the latter is further distinguished by the molecular shape of the constituent molecules. Calamitic phases are formed by cylinder-like mesogens, discotic phases by disk-shaped molecules, and bent-core phases [6,7] by so called banana liquid crystals.

**Figure 1.**
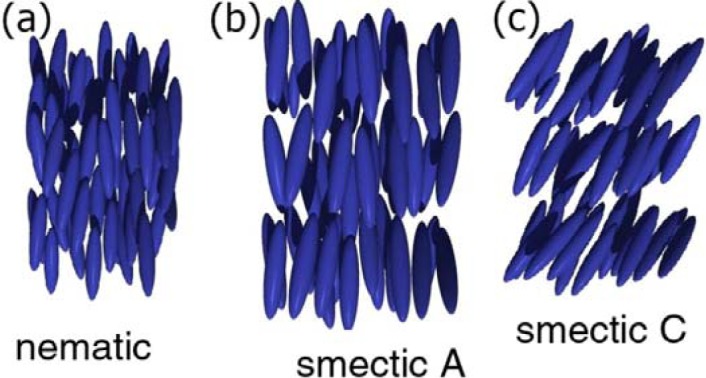
Schematic representation of the (**a**) nematic; (**b**) smectic A (SmA) and (**c**) smectic C (SmC) phase of calamitic (rod-like) mesogens. Molecular order increases on cooling from the left to the right side of the diagram.

Numerous liquid crystal phases can be observed, depending on the order of the constituent mesogens [8]. The simplest of the phases is the nematic phase, which solely exhibits an average orientational order of the long axes of the molecules (called the director **n**), while their centres of mass are isotropically distributed (Figure 1a). The nematic phase is also the one observed at the highest temperature. On cooling, fluid smectic phases are formed, which in addition to orientational order also exhibit a one-dimensional positional ordering, *i.e.*, a layered structure. Within a smectic layer the centres of mass of the mesogens is again isotropically distributed. For the smectic A (SmA) phase the layer normal **k** is parallel to the director **n**, while in the smectic C (SmC) phase it is inclined by a temperature dependent angle called the tilt angle, θ (Figure 1b,c, respectively). Further liquid crystal phases are the hexatic phases, which exhibit short and long range two-dimensional positional order, *i.e.*, an additional feature of order within individual smectic layers.

**Figure 2.**
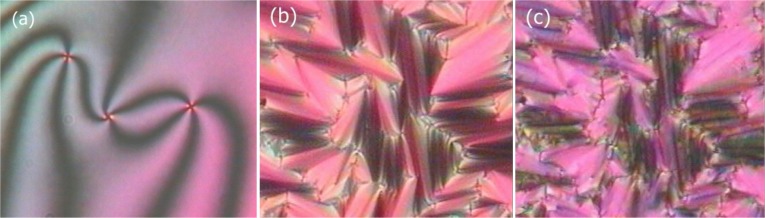
Characteristic polarizing microscopic defect textures of (**a**) the nematic Schlieren texture; (**b**) SmA focal conic and (**c**) broken focal conic texture of the SmC phase. (Reproduced with permission from [8]. Copyright 2003 Wiley-VCH Verlag GmbH&Co. KGaA).

In order to identify different liquid crystalline phases, a variety of experimental techniques is generally employed, among them differential scanning calorimetry (DSC), small angle and wide angle X-ray diffraction (SAXS, WAXS), and texture observation by polarizing optical microscopy (POM) [8]. An example of the latter is shown in Figure 2, where the characteristic defects allow a verification of the phase under investigation, in this case point singularities of a nematic phase (Figure 2a, Schlieren texture), Dupin cyclides of a fluid smectic A phase (Figure 2b, focal conic texture), and the broken fan-shaped texture of SmC (Figure 2c).

### 1.2. Ferroelectric Liquid Crystals [9,10]

Introduction of chirality or “handedness” to liquid crystalline materials leads to exciting changes of the phases. Novel structures are observed, like helical superstructures, novel phases, the so called frustrated phases (Blue Phases [11], Twist Grain Boundary phases [12]), which only occur in chiral systems, are formed, and novel effects can be seen. The latter are, for example, selective reflection leading to photonic bandgaps [13], or extremely fast electro-optic modulation via the electroclinic effect [14,15]. However, the most pronounced observation is that of ferroelectricity in fluid systems [16,17]. Indeed, ferroelectric liquid crystals (FLC) are the only fluid ferroelectrics known to man.

**Figure 3.**
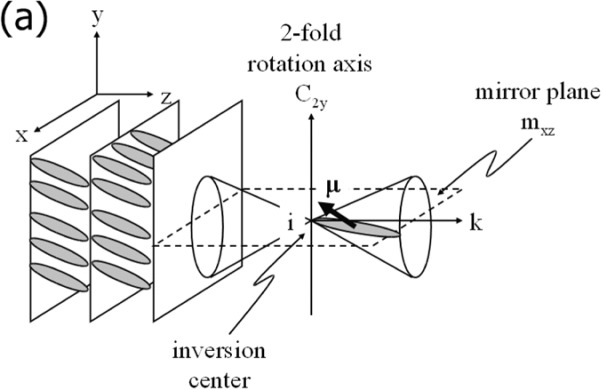

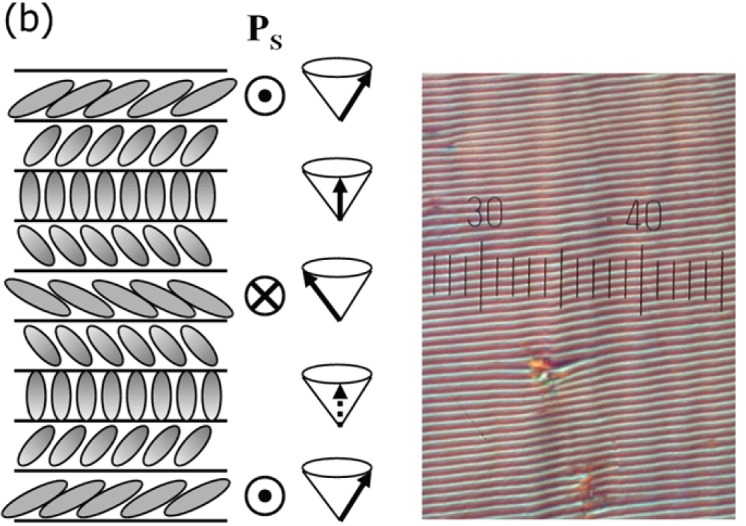
(**a**) Symmetry elements of the achiral SmC phase, explaining the absence of a spontaneous polarization; (**b**) schematic helical superstructure of the chiral SmC* phase (**left**) and associated polarized microscopic image of the equidistant line pattern (**right**). (Reproduced with permission from [8]. Copyright 2003, Wiley-VCH Verlag GmbH&Co. KGaA).

The occurrence of a spontaneous polarization in the SmC* phase can easily be understood by following the symmetry arguments of Meyer [16]. He deduced that all chiral titled smectic phases exhibit a local spontaneous polarization and are thus pyroelectric. In the SmC* phase, as in some others, this polarization is switchable between two stable states, thus ferroelectric. As depicted in Figure 3a, the structure of the achiral SmC phase contains three symmetry elements, namely a two-fold rotation axis, a mirror plane and therefore a resultant inversion centre. Application of those local symmetry elements to a molecular dipole moment μ = (μ_x_, μ_y_, μ_z_) gives μ_i_ = (−μ_x_, −μ_y_, −μ_z_), giving a net dipole moment of the achiral SmC phase of μ_SmC_ = (0, 0, 0), a vanishing spontaneous polarization. For chiral molecules the mirror plane and the inversion centre are lacking and a dipole moment μ = (μ_x_, μ_y_, μ_z_), which is subjected to the only remaining symmetry element, the two-fold rotation axis, becomes μ_rot_ = (−μ_x_, −μ_y_, −μ_z_) and the resultant dipole moment of the chiral SmC* phase is μ_SmC_* = (0, μ_y_, 0). The spontaneous polarization is the sum of the non-vanishing lateral dipole moment components per unit volume. Reversal of an applied electric field switches the spontaneous polarization between two stable states, and the smectic C* structure is ferroelectric.

This is not the whole truth though. For a bulk sample the spontaneous polarization compensates by forming a helical superstructure, and the SmC* phase is more correctly called helielectric. The helical structure of SmC* manifests itself in polarizing microscopy through the appearance of an equidistant line pattern, as shown in the inset of Figure 3b Subjecting the SmC* phase to thin cells with strong surface interactions and suppressing the helical superstructure leads to the so called surface stabilized ferroelectric liquid crystal (SSFLC) [17]. This state is indeed ferroelectric and from a virgin sample a domain structure is observed, which represents the two ferroelectric domains of polarization up and polarization down. The latter is shown in Figure 3b.

### 1.3. Polymer Stabilization [18,19]

Polymer stabilisation of liquid crystals refers to a method first used on nematic and cholesteric phases [20–24] in the development of reflective displays, or electronic paper [25–31]. It is now widely used also for other liquid crystal phases and purposes. A small amount, generally less than 10% by weight, of a photoreactive monomer is mixed into the liquid crystalline phase, while care has to be taken to stay below the solubility limit of the monomer. These photoreactive monomers can be liquid crystalline by themselves, but do not need to be, as long as they exhibit a similar elongated shape and form anisotropy as the liquid crystal host molecules. A minute amount of a photoinitiator, often benzoin methyl ether (BME) is also added to the mixture. The latter will not be incorporated into the actual structure, but is simply present as a catalyst to facilitate the chemical reaction, which will take place when the mixture is subjected to UV illumination.

**Figure 4.**
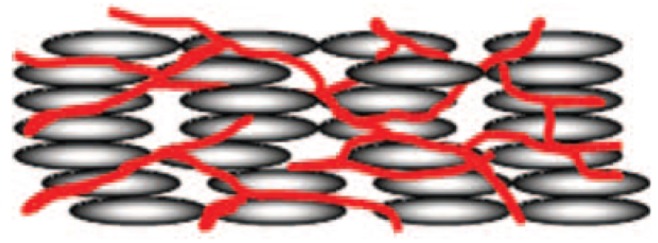
Schematic of a polymer stabilized liquid crystal. The polymer network acts as a template of the self-organised liquid crystalline phase that it was formed in.

The mixture is filled into suitable cells, which promote a certain desired zero electric field alignment. Since the monomers exhibit the same shape anisotropy as the liquid crystal molecules, they will align along the local director of the liquid crystal. UV illumination then causes an open polymer network to be formed by the photoreactive monomers. This polymer network will follow the local director field in which it was formed, thus acting as a template of the liquid crystal structure [32,33] (Figure 4). The network is phase separated from the liquid crystal, and the interaction between both is thus of elastic nature. The liquid crystal may then be forced out of its equilibrium orientation for example by electric or magnetic fields, and on turning the outside stimuli off, the polymer network drives the liquid crystal back to its original director configuration. Other scenarios may be envisioned. The liquid crystal can be washed out by a suitable solvent and be replaced by a different material. A left handed helical polymer network structure may be filled with a right handed cholesteric material of the same or a different pitch [34]. Polymerisation can be carried out in the orthogonal SmA phase, and the transition into the tilted SmC phase can be investigated, a scenario which we will come back to below. In all cases the fundamental idea is the same: a polymer network is formed which stabilises the liquid crystal director configuration in which it was formed. Elastic interactions between the large surface of the polymer network and the liquid crystal will aim to drive the system back into its equilibrium orientation.

**Figure 5.**
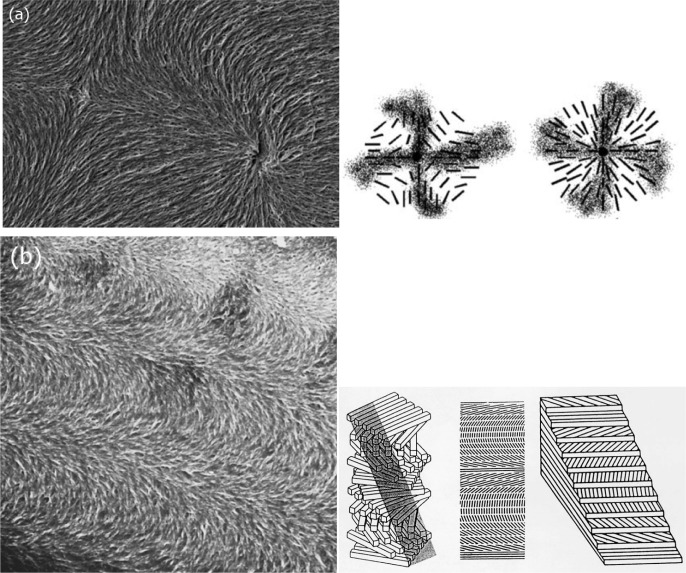

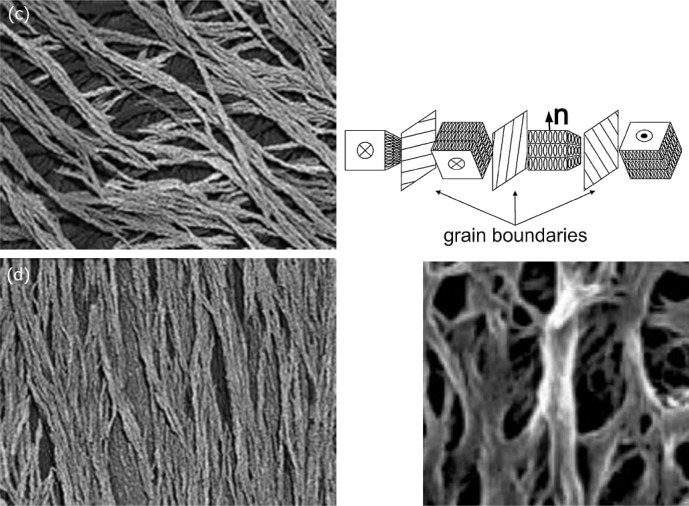
Exemplary scanning electron microscopy (SEM) images of polymer networks formed in different liquid crystalline phases: (**a**) nematic s = ±1 defect pair (**top**) and schematic director field (**bottom**); image side length ~50 μm (Reproduced with permission from [35]. Copyright 2013 Royal Society of Chemistry); (**b**) helical superstructure of the chiral nematic, N* phase (**top**), which can be illustrated by an oblique cut called a Bouligand cut (**bottom**); image side length ~50 μm (Reproduced with permission from [33]. Copyright 1997 American Physical Society); (**c**) different director orientation in SmA* twist grain boundary (TGB) blocks (**top**) and schematic representation (**bottom**); image side length ~5 μm. (Reproduced with permission from [36]. Copyright 2009 Royal Society of Chemistry) and (**d**) well oriented SmA phase (**left**) and close-up (**right**); image side length ~5 μm. The material used is the well known RM257 from Merck, and the liquid crystal has been removed by a suitable solvent after polymerization. For the SEM investigations the polymer network was then coated with a very thin layer of gold.

A few examples of polymer networks formed in different liquid crystals are presented in Figure 5 [33,35–37]. The images are taken by scanning electron microscopy, after coating the polymer network with a thin layer of gold. In part (a) a s = +1 point defect is shown (left), which clearly represents the expected director field from a defect of a Schlieren texture [35] (right) as introduced in Figure 2a. Figure 5b demonstrates the helical superstructure of the cholesteric or chiral nematic phase by an oblique cut through the helix from the top to the bottom substrate plates. The pitch was adjusted to 10 μm so that three half turns of the helix are visible in the 15 μm thick cell [33] (left). The imaged structure is equivalent to a so called Bouligand cut (right), which is used to demonstrate helical superstructures in biological systems. Similarly, the individual smectic blocks of a twist grain boundary (TGB) phase can be imaged [36] (Figure 5c left), including the discontinuous twist which is mediated via boundaries of screw dislocations (right). Also such structures can be found in biological systems. In the SmA phase the polymer network is well oriented along the director of a uniform sample (Figure 5d left) [36]. It can be seen that the individual features of the polymer strands are on average about 0.1 μm in size (Figure 5d right). The size of the voids depends on various preparation conditions, such as monomer concentration, polymerisation temperature, and UV dose [29]. In general, the polymer network formation process is completed within about 10–15 min.

## 2. Effects on SmC* Material Parameters

The first polymer stabilized ferroelectric liquid crystal (PSFLC) was made by Hikmet and Lub [38] from Philips in 1995, in an effort to overcome the mechanical problems of surface stabilized ferroelectric liquid crystals. Samples were polymerized in the SmC* phase and an oriented polymer network along the smectic layer normal was observed. It was found [39] that polymer network stabilization has a considerable influence on the electro-optic performance of FLCs and the physical parameters as compared to the non-stabilized sample.

### 2.1. Tilt Angle and Spontaneous Polarization

**Figure 6.**
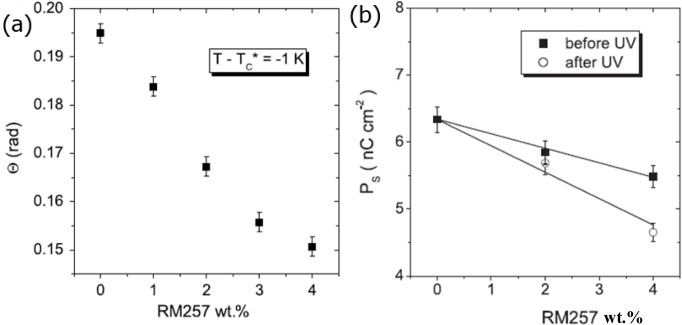
Effect of the polymer network on the principle order parameters of the SmA*–SmC* transition, (**a**) tilt angle (Reproduced with permission from [42]. Copyright 2008, Institute of Physics); and (**b**) spontaneous polarization (Reproduced with permission from [44]. Copyright 2009 Institute of Physics). Both quantities decrease for increasing polymer network concentration.

**Figure 7.**
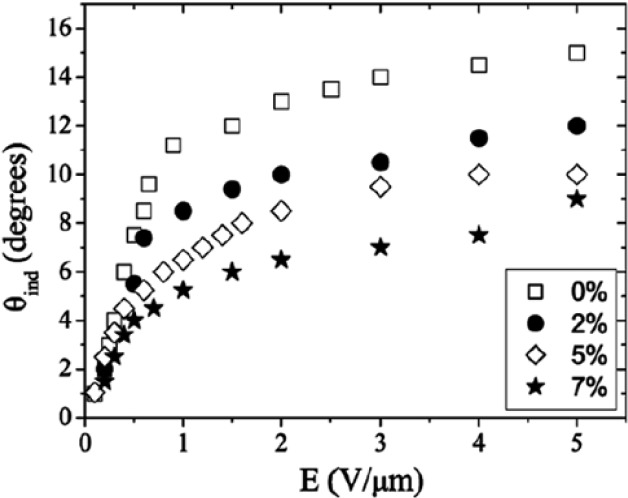
Effect of the polymer network on the electroclinically induced tilt angle in the vicinity of the SmA*–SmC* transition. The electroclinic effect is reduced for increasing polymer content. (Reproduced with permission from [43]. Copyright 2008 Taylor & Francis).

The two most fundamental parameters of the SmC* phase are the tilt angle and the spontaneous polarization. The former is the primary order parameter for the transition between the non-tilted, paraelectric SmA* and the tilted, ferroelectric SmC* phase, while the spontaneous polarization represents the secondary order parameter of the transition, as it is coupled to the tilt angle. While maintaining their general temperature dependence of increasing values for decreasing temperatures, both the tilt angle and the polarization decrease for increasing polymer concentration [40–42]. This is demonstrated in Figure 6a,b, respectively. The reason for this behaviour can be found in regions in the vicinity of the network that are strongly dominated by the polymer, thus not switching at all, or at least only partially, in contrast to the saturated switching of the bulk regions. There is thus a decrease of the effective tilt angle and the polarization. The polymer dominated regions become more and more pronounced with increasing polymer concentration, and the two prime parameters of the SmC* phase decrease for increasing polymer content. The electroclinic effect, describing an induced tilt angle in the very close vicinity of the SmA*–SmC* transition is in our case practically not affected by the process of polymer stabilization, most likely due to the fact that it is very small and only observed over a narrow temperature regime. The situation appears to be different for large electroclinic effect materials, as shown by Petit *et al.* [43] in Figure 7. Here the increase in polymer network density causes a decrease of the electroclinically induced tilt angle, as would be expected. The fact that the respective investigations were carried out on short-pitch FLCs in contrast to the normally investigated surface stabilized FLCs may also play a role for the interpretation of these results.

### 2.2. Landau Coefficients and Interaction Parameter

In physical terms, this behaviour can be described by a generalized Landau model of the SmA* to SmC* transition [45,46], which is extended by an additional factor, describing the interaction between the liquid crystal and the polymer network. In a non-helical FLC device subjected to an electric field, E, the difference in the free energy density between the SmA* and SmC* phase, g–g_0_, is given by:

$$g-g_{0}=\frac{1}{2}\alpha (T-T_{C})\Theta^{2}+\frac{1}{4}b\Theta^{4}+\frac{1}{6}c\Theta^{6}+\frac{P^{2}}{2\varepsilon_{0}\chi_{0}}-C\Theta P-\frac{\Omega P^{2}\Theta^{2}}{2}-PE$$ (1)

where Θ is the tilt angle, *P* is the total polarisation, *a* = α(T-T_C_), *b* and *c* are the first three Landau expansion coefficients, C is the bilinear coupling coefficient, χ_0_ is the high frequency dielectric susceptibility in the direction of the electric field, Ω is the biquadratic coupling coefficient and T_C_ is the transition temperature of the related achiral SmA to SmC transition. It should be noted that the Landau description is only valid in the vicinity of the phase transition. Nevertheless, this is the region where the significant changes are observed. For PSFLC systems an additional term is required in the generalised Landau model to take into account the interaction between the polymer network and the liquid crystal. Employing the simple model of Li *et al.* [47] the interaction between the polymer network and the liquid crystal acts to restore the local liquid crystal director to the orientation of the polymer network. They introduced an elastic coupling interaction term, $\frac{1}{2}W_{P}\sin^{2}\Theta \approx \frac{1}{2}W_{P}\Theta^{2}$, where *W_P_* is the interaction coefficient between the polymer network and the liquid crystal. The free energy density then reads as:

$$g-g_{0}=\frac{1}{2}\alpha (T-T_{C})\Theta^{2}+\frac{1}{2}W_{P}\Theta^{2}+\frac{1}{4}b\Theta^{4}+\frac{1}{6}c\Theta^{6}+\frac{P^{2}}{2\varepsilon_{0}\chi_{0}}-C\Theta P-\frac{\Omega P^{2}\Theta^{2}}{2}-PE$$ (2)

Minimisation of the free energy density (Equation (2)) with respect to the total polarisation P leads to the relation [48,49]:

$$P=\frac{C\Theta +E}{\frac{1}{\varepsilon_{0}\chi_{0}}-\Omega \Theta^{2}}$$ (3)

Simultaneous fitting of the total polarisation as a function of tilt angle with respect to varying applied electric field amplitude allows the determination of *C*, χ_0_ and Ω. Minimisation of Equation (2) with respect to Θ and resubstitution of Equation (3) leads to a temperature-tilt relationship of:

$$T(\Theta ,E)=T_{C}-\frac{1}{\alpha }\left[W_{P}+b\Theta^{2}+c\Theta^{4}-\frac{\left(C\Theta +E\right)\left(\frac{C}{\varepsilon_{0}\chi_{0}}+\Omega \Theta E\right)}{\Theta {\left(\frac{1}{\varepsilon_{0}\chi_{0}}-\Omega \Theta^{2}\right)}^{2}}\right]$$ (4)

**Figure 8.**
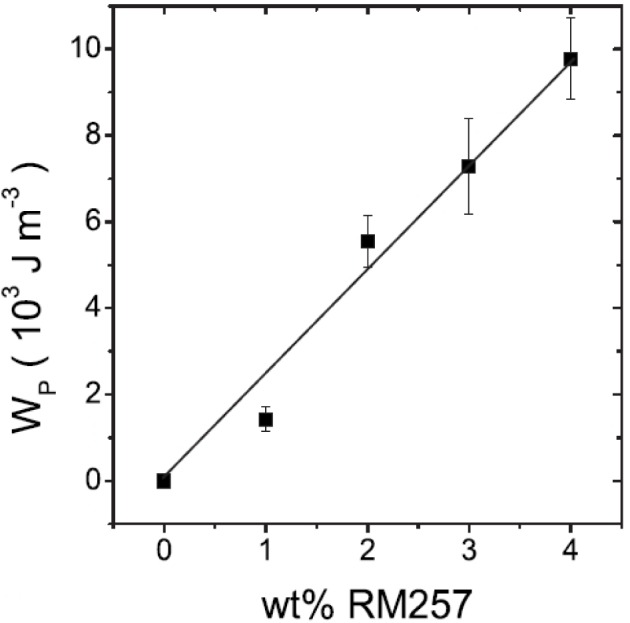
For small concentrations the elastic coupling coefficient between polymer network and liquid crystal increases linearly with increasing network density. (Reproduced with permission from [50]. Copyright 2008 American Physical Society).

For T_C_ ≈ T_C_* Equation (4) can be directly employed to determine α, b, c and W_P_ via a second set of simultaneously fitted curves T(Θ,E), using the previously determined parameters C, χ_0_ and Ω. One finds that the Landau coefficients are largely unaffected by the introduction of the polymer network, while the elastic coupling coefficient linearly increases for increasing polymer concentration [42,50] (Figure 8). A more thorough analysis, taking into account the physical dimensions of the network shows, that the elastic coupling coefficient depends on polymer concentration and the penetration depth of the elastic forces into the bulk of the liquid crystal. A linear increase of the elastic coupling coefficient with values of the same order of magnitude was also reported by Petit *et al.* [51].

### 2.3. Response Times and Effective Viscosity

**Figure 9.**
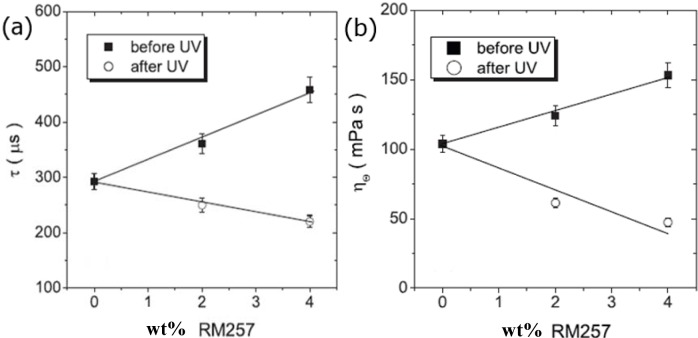
(**a**) The response time after formation of the polymer network decreases for increasing network density and (**b**) for the effective viscosity a comparable trend is observed. (Reproduced with permission from [44]. Copyright 2009 Institute of Physics).

In the ferroelectric SmC* phase the response times exponentially increase for decreasing temperature, which is a plain viscosity effect when observed at equal applied voltages. This behaviour is not changed for PSFLCs. It is generally found that mixing the monomer into the FLC material increases the response times the more monomer is added. This effect is most likely due to the relatively large size of the monomers, despite themselves being liquid crystalline over a certain range of temperature, with the monomer increasing the viscosity of the FLC-monomer mixtures. Subsequent UV polymerization of the monomers and formation of the polymer networks then reduces the effective viscosity and the response times of the PSFLC become shorter, even shorter than those observed for the neat FLC material [44]. It is further found that after polymerization the response times decrease for increasing polymer network concentration, as depicted in Figure 9a. Since the response time τ is directly proportional to the effective viscosity η via τ = η/P_S_E, the equivalent trend is also observed for the viscosity as a function of polymer network content (Figure 9b). Such behaviour has been reported by several authors [52–55], but it should be pointed out, that results of the opposite behaviour have also been published, *i.e.*, an increase in viscosity with increasing polymer concentration [56].

### 2.4. Dielectric Spectroscopy

**Figure 10.**
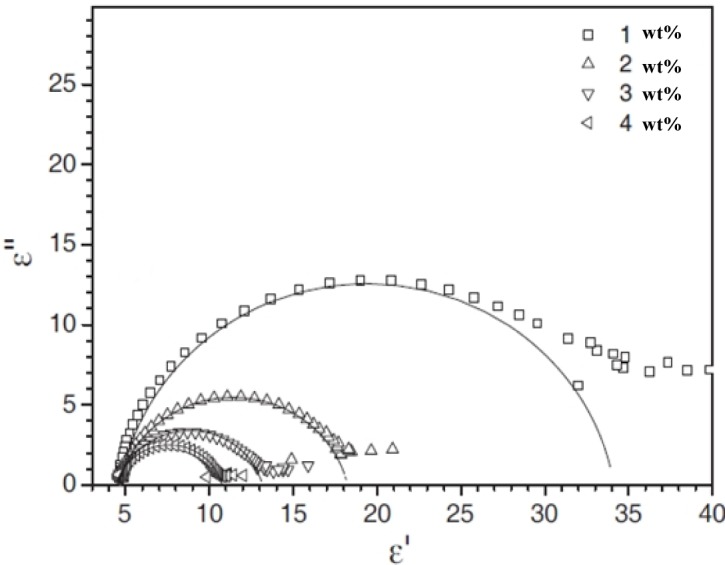
Cole-Cole plot, ε″ *versus* ε′, of the dielectric properties for varying polymer network concentration. (Reproduced with permission from [58]. Copyright 2009 EDP Sciences).

**Figure 11.**
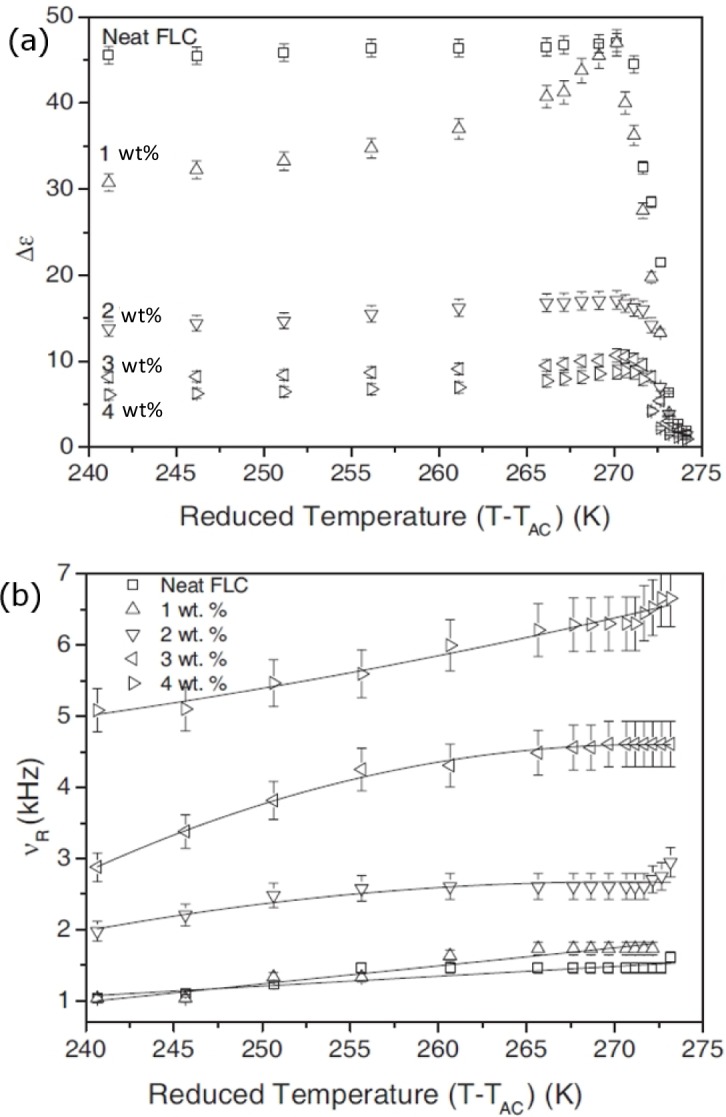
(**a**) The dielectric strength of the Goldstone mode is suppressed for increasing polymer concentration; while (**b**) the relaxation frequency increases with increasing network density. (Reproduced with permission from [58]. Copyright 2009 EDP Sciences).

The ferroelectric SmC* phase exhibits two major collective dielectric modes, the Goldstone mode and the softmode. The former is related to tilt fluctuations on the cone, while the latter is due to tilt fluctuations changing the value of the tilt angle. It should be noted that the effect of a polymer network on the softmode is in general relatively small [57], while pronounced effects are observed on the Goldstone mode. We will thus focus our discussion on the Goldstone mode, and the collective fluctuations of the director on the tilt cone [58]. Dielectric spectroscopic data is often illustrated in a frequency independent plot of the dielectric absorption ε″ as a function of the dielectric permittivity ε′, the so called Cole-Cole plot, as it is shown in Figure 10 for different polymer network concentrations. Already here it can be seen that an increase of polymer network density suppresses the Goldstone mode, or more precisely, it decreases the dielectric strength (Δε = ε_0_ − ε_∞_) for all temperatures in the SmC* phase (Figure 11a), while the relaxation frequency ν_R_ is increased (Figure 11b). This general behaviour has been reported by numerous authors [53,57–60] and seems to be noncontroversial. It can be explained by the reduction of collective fluctuations due to an increasing density of polymer network, and thus an increase of elastic interactions between liquid crystal and network. The effect of the polymer network is equivalent to that of an application of an electric bias field [58]. The dielectric strength decreases for increasing polymer concentration, because more and more liquid crystal molecules are elastically coupled to the polymer, reducing their collective fluctuations. The relaxation frequency is proportional to the effective elastic constant K_eff_ and inversely proportional to the effective viscosity, ν_R_~K_eff_/η_eff_, and thus displays a behaviour as expected with increasing network content.

**Figure 12.**
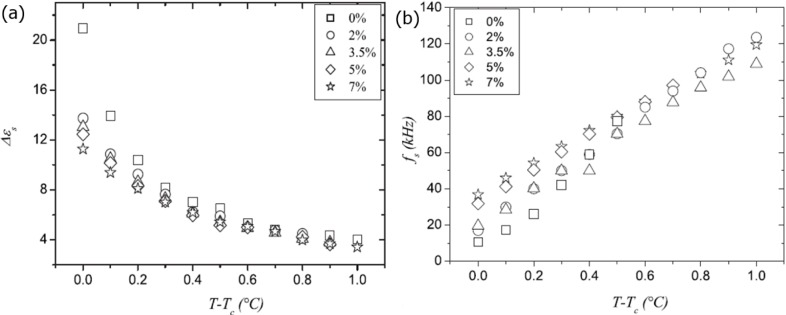
(**a**) Also the softmode dielectric strength is slightly suppressed for increasing polymer concentration; while (**b**) the softmode relaxation frequency slightly increases. (Reproduced with permission from [57]. Copyright 2009 American Physical Society).

As mentioned above, the effect of the polymer network on the softmode of a ferroelectric liquid crystal appears to be much less pronounced [57]. The softmode appears at frequencies above the Goldstone mode, and also here a slight decrease of the dielectric strength is observed for increasing network density at equal reduced temperatures, as depicted in Figure 12a, while the relaxation frequency slightly increases (Figure 12b).

In the context of display applications of FLCs, also the stabilization of the so called V-shaped switching mode, which basically exhibits a thresholdless, linear, hysteresis-free electro-optic response to an applied electric field, is of much interest [61,62]. Stabilizing the texture and electro-optic response of such V-shaped switching has been the focus of numerous papers [47,63–66] on polymer stabilized antiferroelectric liquid crystals. Hysteresis may be observed in certain PSFLC materials, especially for low monomer concentrations, which limits application aspects of these materials. Nevertheless, quite often the hysteresis is small and not of significant relevance.

## 3. Antiferroelectric Materials

SmC* liquid crystals can not only exhibit a ferroelectric structure with all molecules tilted in the same direction (synclinic), but also an antiferroelectric modification, where molecules of adjacent smectic layers are tilted in the opposite direction (anticlinic, disregarding any helical superstructure in a simplified view). The electro-optic switching of these systems is monostable, as compared to the bistable switching of the ferroelectric phase. The antiferroelectric phase switches from the stable E = 0 V state to either one of the ferroelectric states, depending on the polarity of the applied voltage.

### 3.1. Calamitic Antiferroelectrics

**Figure 13.**
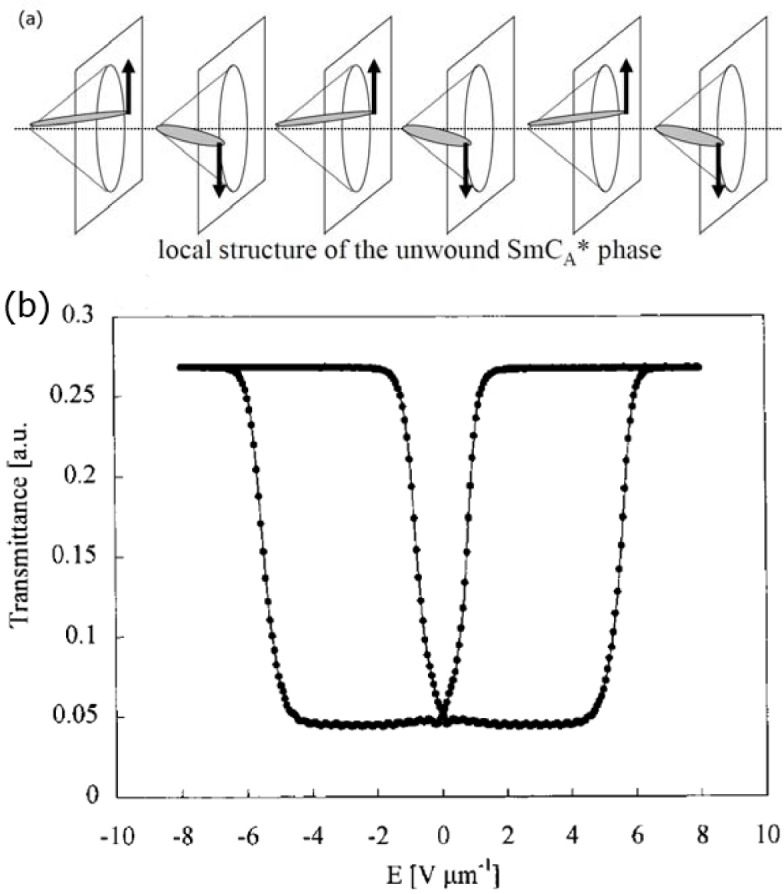
(**a**) Schematic structural drawing of an antiferroelectric liquid crystal, without the helical superstructure (Reproduced with permission from [8]. Copyright 2003 Wiley-VCH) and (**b**) standard electro-optic response of the SmC_A_* phase ((Reproduced with permission from [71]. Copyright 2001 American Chemical Society).

**Figure 14.**
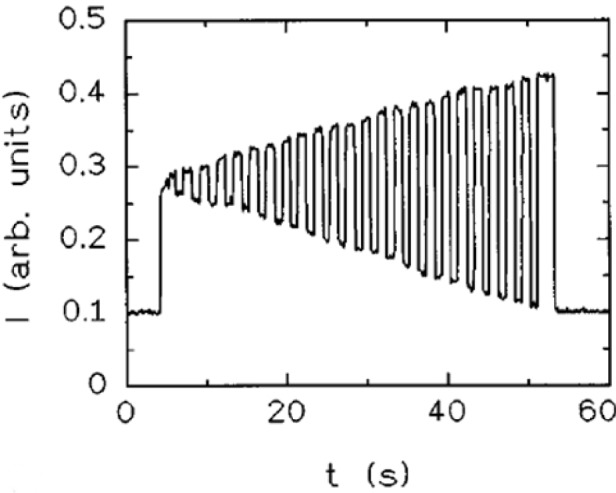
Electro-optic response of a polymer stabilized antiferroelectric liquid crystal, which allows for easier gray scale generation. (Reproduced with permission from [70]. Copyright 1996 American Institute of Physics).

Antiferroelectricity in liquid crystals is attributed to have been discovered first for rod-like, elongated molecules [67–70]. The director configuration in its simple form is depicted in Figure 13a, together with the standard electro-optic response (Figure 13b). Introduction of a polymer network makes the switching transition between antiferroelectric to ferroelectric states more continuous, which allows for easy gray scale generation [71,72] (see Figure 14), an advantage of the antiferroelectric SmC*_A_ phase over the ferroelectric SmC* phase, when display applications are considered. Of special interest for display applications are the orthoconic antiferroelectric liquid crystal materials, systems with a tilt angle of 45°, or very close to that value [73,74]. These systems exhibit a very good dark state due to the zero birefringence at these tilt angles. Also in this case, polymer stabilization was used to improve the mechanical stability [75].

In general, like for ferroelectric liquid crystals, also in antiferroelectric phases the polymer network has an influence on the fundamental material parameters. The contrast of the switching process reduces for increasing polymer concentration, as does the spontaneous polarization. This is due to an effective tilt angle, which is smaller than that observed for the neat liquid crystal, as an increasing amount of molecules contributes less and less to the switching process for increasing network content, due to elastic interactions between liquid crystal and polymer network.

### 3.2. Bent-Core Antiferroelectrics

Bent-core mesogens form quite remarkable liquid crystal phases [6,7], in so far as structural chirality and chiral effects can be observed from achiral molecules [76]. Alternatively, addition of an achiral bent-core material to a chiral matrix has been shown to increase chirality, for example through the decrease of the cholesteric pitch [77], the occurrence [78] or widening [79] of a Blue phase, and the increase of the chiral bilinear tilt-polarization coupling coefficient [80,81]. As neat materials, bent-core phases mostly exhibit antiferroelectric properties. One of the largest obstacles in their use for applications is the fact that bent-core phases are notoriously difficult to orient uniformly, thus diminishing their electro-optic response quality. A recent study [82] of the switching performance of polymer stabilized antiferroelectric bent-core phases has revealed a large temperature region, improved alignment ability, and enhanced contrast for such systems. In addition, it was found that the response times decrease for increasing polymer network content, in agreement with results on polymer stabilized ferroelectric liquid crystals.

## 4. Summary

The basic principles of polymer stabilization of ferroelectric liquid crystals were outlined, and their influence on the most important physical parameters characterized. It was shown that both the tilt angle and the spontaneous polarization effectively decrease with increasing polymer concentration. This was explained by the liquid crystal being partially dominated by the network, not contributing to the switching process, due to elastic binding to the polymer. A Landau analysis provides evidence for this interpretation, because an introduced interaction parameter between liquid crystal molecules and stabilizing polymer network increases linearly with increasing network density at small polymer concentrations. The response times of the polymer stabilized systems are found to be shorter than those of the neat liquid crystal, and decrease with increasing network concentration. This also implies that the effective viscosity of the stabilized system decreases. While the process of polymer stabilization does not exhibit a pronounced effect on the softmode dielectric relaxation, it is clearly observable in the lower frequency Goldstone mode relaxation. The Goldstone mode in general gets suppressed and the dielectric strength reduces with increasing polymer concentration, while the relaxation frequency increases. This behaviour conforms with the interpretation above: elastic binding of the molecules to the network reduces director fluctuations, while it enhances their relaxation frequency. A similar effect of the polymer network as on the parameters of the ferroelectric SmC* phase, can be observed for the antiferroelectric state, independent of whether this state is formed by calamitic or by bent-core mesogens.
